# Mucocele of the Appendix: A Case Report and Review of Literature

**DOI:** 10.7759/cureus.40168

**Published:** 2023-06-09

**Authors:** Arshadullah Khan, Renad S AlSubaie, Ali A Almohammed Saleh

**Affiliations:** 1 Oncology and Breast Oncoplastic Surgery, Al Ahsa Hospital, Al-Hofuf, SAU; 2 Medicine, King Faisal University, Al-Hofuf, SAU

**Keywords:** appendix, mucocele, laparoscopy, appendicular mucocele, mucinous cystadenoma

## Abstract

An appendiceal mucocele is a rare disease characterized by the dilation of the appendix lumen with mucus accumulation. Although this disease is often found incidentally during appendectomy, it is crucial to differentiate it from acute appendicitis preoperatively to select adequate surgical management. We present a case of a 31-year-old male, medically free, with right-sided abdominal pain associated with nausea and vomiting. He was diagnosed with appendiceal mucocele and underwent laparoscopic appendectomy. The absence of a distinct clinical presentation and biochemical parameters necessitates a comprehensive and collaborative diagnostic approach for mucocele of the appendix. Achieving an accurate diagnosis prior to surgery is imperative to ensure the appropriate surgical technique is chosen, thereby minimizing the risk of serious intraoperative and postoperative complications such as pseudomyxoma peritonei.

## Introduction

Appendectomy is considered the most common emergency surgical operation performed worldwide [1]. Although acute appendicitis is the predominant underlying cause behind this procedure, many other appendicular diseases have been recognized [2]. Appendiceal mucocele is a rare disease with an incidence of 0.2%-0.7% of all appendectomy specimens [3]. It was first described in 1842 by Rokitansky and characterized by the dilation of the appendix lumen with mucus accumulation [4,5]. There are four known histological types of appendiceal mucocele: retention cysts, cystadenomas, cystadenocarcinomas, and mucosal hyperplasia [6]. Clinically, patients affected by this disease do not have specific clinical features, and it is frequently found incidentally during appendectomy [7]. The lack of distinctive clinical features can lead to the misidentification of this condition as acute appendicitis, resulting in incorrect treatment. Thus, it is imperative to differentiate between these two pathologies prior to surgery to ensure appropriate surgical management. Inadequate treatment of an appendiceal mucocele may cause its advancement into pseudomyxoma peritonei, a condition resulting from the release of epithelial cells into the peritoneal cavity [8]. We hereby report a case of a 31-year-old male patient, medically free, who presented to the clinic complaining of recurrent right-sided abdominal pain for 15 days. The pain was associated with nausea and vomiting. There was no history of weight loss or loss of appetite. The patient was diagnosed by computed tomography (CT) as a case of appendiceal mucocele and scheduled for laparoscopic appendectomy. He was discharged on the fourth day postoperatively with no local or systemic complications.

## Case presentation

**Figure 1:**
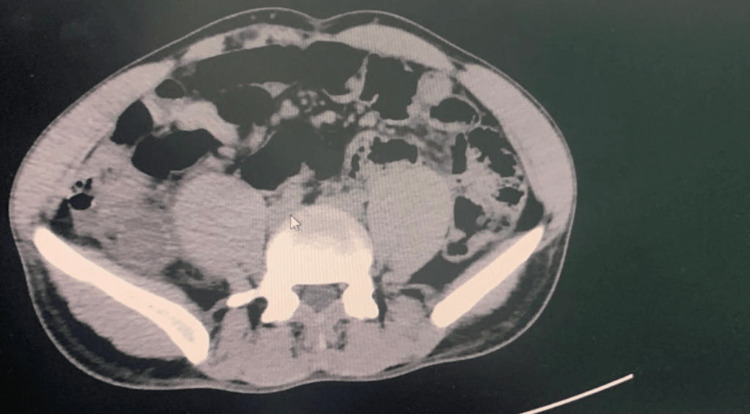
Contrast-enhanced computed tomography of the abdomen showing a tubular mass in the appendiceal area.

**Figure 2:**
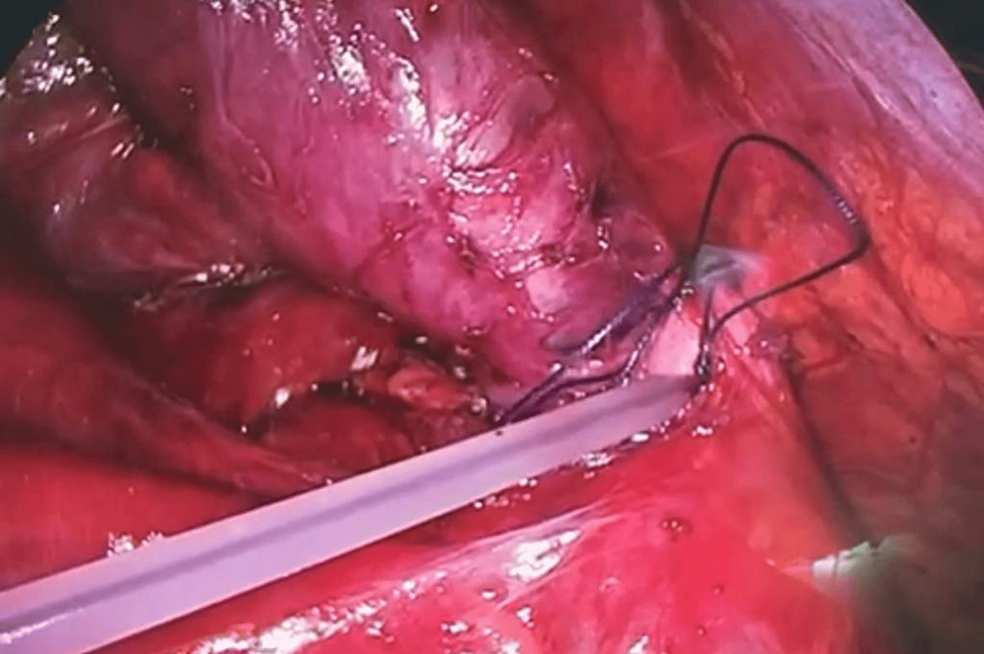
Intraoperative finding (enlarged appendix).

A 31-year-old male patient, medically free, presented to the clinic complaining of lower right-sided abdominal pain for 15 days associated with nausea and vomiting. The pain was recurrent, localized, and non-radiating and was not relieved by analgesics. There was no relieving or exacerbating factors of the pain as well as no history of loss of appetite or documented weight loss. There was neither a history of hematemesis nor melena. His surgical, medical, and family histories were insignificant. He is a non-smoker and non-alcoholic and has no history of drug abuse. The patient was afebrile and hemodynamically stable. Abdominal examination revealed mild tenderness over the right iliac fossa (RIF), and rebound tenderness was elicited. The digital rectal examination revealed a normal anal tone and an empty rectum with no signs of bleeding. His laboratory workup including complete blood count, serum creatinine, and liver function tests was all within normal limits. Abdominal and pelvis computed tomography (CT) scan with oral and intravenous contrast was performed. It demonstrated a fluid-filled tubular mass in the RIF. The wall of the lesion shows specks of calcification; fat infiltration was also seen. No enlarged or suspicious regional mesenteric lymphadenopathy was detected (Figure 1). The CT findings were compatible with a mucocele of the appendix, and a laparoscopic appendectomy was scheduled. Intraoperatively, a pale mass of the appendix with dimensions 8 × 2.5 × 2.5 cm but without perforation was discovered in the RIF. The appendiceal lumen dilated with a thinned-out wall, and a grayish-yellow mucus collection was also seen (Figure 2). The cecum, terminal ileum loops, and bilateral adnexa were grossly normal. Histopathological examination of the appendix showed wide mucosal denudation, lymphocytes, plasma cells, and eosinophils in the wall of the appendix. No malignant cells were found in the resected ileocolic lymph nodes. All margins were free from malignant cells. The patient was discharged home on the fourth postoperative day and advised to follow up after 10 days. He had no local or systemic complications.

## Discussion

An appendicular mucocele is a rare condition characterized by the presence of groups of mucus-filled lesions that obstruct the ileocecal appendix. This condition is relatively uncommon. However, it is important to note that appendicular mucocele is a significant contributor to appendiceal tumors, representing approximately 8% of all cases [9]. Despite its rarity, mucinous cystadenoma of the appendix is the most prevalent type of benign neoplasm affecting the appendix. It is estimated to occur in approximately 0.6% of all appendectomy specimens [10]. Mucinous cystadenoma is often associated with abdominal pain, which is the most common presenting symptom. However, it is important to note that about a quarter of patients with this condition do not experience any symptoms, and the diagnosis is made incidentally. It is worth noting that appendiceal mucocele can mimic other medical conditions such as acute appendicitis, appendicular plastron, or cecal tumor [11,12]. Besides abdominal pain, other symptoms such as hemorrhage, intussusception, and local invasion into nearby structures have been reported in some cases [13-15]. It can be categorized based on the histological characteristics of the lumen obstruction. There are two types of mucocele: simple mucocele and hyperplastic mucocele. Simple mucocele can result from inflammatory, obstructive, or retention cysts, leading to degenerative epithelial changes that cause the appendix to become obstructed and distended. In contrast, hyperplastic mucocele is due to the hyperplastic growth of the appendix or cecal mucosa. Furthermore, mucinous cystadenoma is a type of neoplasm that affects the appendix, characterized by dysplastic epithelium.

Mucinous cystadenocarcinoma is a more severe form of appendiceal mucocele that features high-grade cellular dysplasia and stromal invasion, along with muscularis mucosae involvement. The classification of appendiceal mucocele is essential in making an accurate diagnosis and determining the appropriate treatment approach [16]. The diagnosis of appendiceal mucocele preoperatively is challenging. Abdominal ultrasonography (US) is used to be the first-line diagnostic method in any patient presenting with abdominal pain. In the case of mucocele of the appendix, it can be distinguished between benign and malignant mucoceles and usually shows a well-encapsulated cystic lesion containing onion skin-like layers with variable echogenicity [17]. A CT scan of the abdomen is the best radiological imaging, and it is required to confirm the diagnosis. Appendiceal mucocele at CT scan appears as a well-encapsulated cystic lesion with variable wall thickness. Furthermore, mural calcification has been reported with a high frequency reaching up to 50% in patients with appendicular mucinous cystadenomas [18,19]. A few articles in the literature have reported appendiceal mucinous cystadenoma coexisting with appendicular carcinoid tumors; therefore, colonoscopy sometimes is mandatory to rule out associated colon tumors [20]. Endoscopy with targeted biopsy of the appendix is helpful in the preoperative diagnosis, but it is quite difficult due to the narrowness of the appendiceal lumen. However, the disease usually is an incidental finding during surgery, and the diagnosis is achieved with a definitive histopathological assessment of the surgical specimen [21]. Surgical resection of the appendiceal mucocele is the preferred treatment, and selecting which surgical method to use is critical. The best surgical approach to deal with mucocele of the appendix is controversial, and laparotomy has been recommended by many authors to avoid rupture of the mucocele and seeding of trocar sites [22]. However, laparoscopic surgery provides the advantages of good exposure and evaluation of the entire abdominal cavity, as well as more fast recovery with the avoidance of a large incision and a better cosmetic result [23]. If a laparoscopic approach is adopted, care must be taken intraoperatively not to cause content spillage leading to the formation of pseudomyxoma peritonei [24]. Because in the presence of pseudomyxoma peritonei, a surgical aggressive attitude is required, which includes right hemicolectomy, omentectomy, removal of all peritoneal mucin masses, and intraperitoneal chemotherapy [25]. However, our patient was managed by laparoscopic technique successfully with a satisfactory outcome.

## Conclusions

Appendiceal mucocele is a rare condition having a clinical picture that resembles acute appendicitis. The diagnosis of this condition involves a lengthy, multidisciplinary diagnostic process, as it lacks specific clinical features and biochemical parameters. Accurate preoperative diagnosis is essential for selecting appropriate surgical techniques and avoiding potentially severe intraoperative and postoperative complications.
